# Impact of the degree of worsening renal function and B-type natriuretic peptide on the prognosis of patients with acute heart failure

**DOI:** 10.3389/fcvm.2023.1103813

**Published:** 2023-04-03

**Authors:** Dongfang Zhao, Lijie Gu, Wenqian Wei, Dan Peng, Man Yang, Weijie Yuan, Shu Rong

**Affiliations:** Nephrology, Shanghai General Hospital, Shanghai Jiao Tong University School of Medicine, Shanghai, China

**Keywords:** acute heart failure, B-type natriuretic peptide, worsening renal function, mortality, prognosis

## Abstract

**Background:**

The impact of the degree of worsening renal function (WRF) and B-type natriuretic peptide (BNP) on the prognosis of patients with acute heart failure (AHF) is still debatable. The present study investigated the influence of different degrees of WRF and BNP levels at discharge on 1-year all-cause mortality in AHF.

**Methods:**

Hospitalized AHF patients diagnosed with acute new-onset/worsening of chronic heart failure (HF) between January 2015 and December 2019 were included in this study. Patients were assigned into high and low BNP groups based on the median BNP level at discharge (464 pg/ml). According to serum creatinine (Scr) levels, WRF was divided into non-severe WRF (nsWRF) (Scr increased ≥0.3 mg/dl and <0.5 mg/dl) and severe WRF (sWRF) (Scr increased ≥0.5 mg/dl); non-WRF (nWRF) was defined as Scr increased of <0.3 mg/dl). Multivariable cox regression was used to evaluate the association of low BNP value and different degrees of WRF with a all-cause death, as well as testing for an interaction between the two.

**Results:**

Among 440 patients in the high BNP group, there was a significant difference in WRF on mortality (nWRF vs. nsWRF vs. sWRF: 22% vs. 23.8% vs. 58.8%, *P *< 0.001). Yet, mortality did not significantly differ across the WRF subgroups in the low BNP group (nWRF vs. nsWRF vs. sWRF: 9.1% vs. 6.1% vs. 15.2%, *P* = 0.489). In multivariate Cox regression analysis, low BNP group at discharge (HR, 0.265; 95%CI, 0.162–0.434; *P* < 0.001) and sWRF (HR, 2.838; 95%CI, 1.756–4.589; *P* < 0.001) were independent predictors of 1-year mortality in AHF.There was a significant interaction between low BNP group and sWRF(HR, 0.225; 95%CI, 0.055–0.918; *P* < 0.05).

**Conclusions:**

nsWRF does not increase the 1-year mortality in AHF patients, whereas sWRF does. A low BNP value at discharge is associated with better long-term outcomes and mitigates the adverse effects of sWRF on prognosis.

## Introduction

Acute renal impairment is a common complication in patients with acute heart failure (AHF), also known as type I cardiorenal syndrome (1). In patients with type I cardiorenal syndrome, acute renal impairment is assessed as worsening renal function (WRF) and is often defined as a 0.3 mg/dl increase in serum creatinine (Scr) level compared to admission (2). The estimated incidence of WRF in AHF is 25%–33% (3). WRF has traditionally been associated with worse long-term mortality; however, recent studies have challenged this association (4–6). The pathogenic mechanism of WRF in AHF is complex, and these opposing conclusions may stem from different pathophysiologic processes. Decreased renal perfusion, i.e., impaired renal “preload” was previously thought to be the main etiology of WRF (7, 8). However, renal congestion from volume overload may also contribute to WRF (9). Studies have shown that elevated central venous pressure is associated with the occurrence of WRF and that fluid volume status is inseparable from the occurrence of WRF (10, 11). More recently, several studies suggested that AHF patients with successful decongestion at discharge have favorable prognosis and that fluid volume status affects the relationship between WRF and prognosis (12, 13). Thus, adequately evaluating a patient's volume status in the setting of WRF can help understand its prognosis in AHF.

B-type natriuretic peptide (BNP) is a well-established biomarker in AHF, whose secretion is mainly dependent on ventricular volume expansion (14). Therefore, BNP levels may help physicians interpret the patients' volume status. Yet, the effect of different degrees of WRF on AHF prognosis under different BNP at discharge has not been studied. In this study, we divided WRF into two levels, nsWRF and sWRF, according to different degrees of Scr elevation during hospitalization to evaluate the effect of BNP levels at discharge and different degrees of WRF, as well as testing for an interaction between the two on mortality in AHF.

## Methods

### Patients

Patients diagnosed with acute new-onset/worsening of chronic heart failure (HF) in Shanghai General Hospital between January 2015 and December 2019 were included in this study. Demographic characteristics, medical history, physical examination results, laboratory indicators, etc., were collected at admission, during hospitalization, and at discharge. AHF was diagnosed based on the 2021 European Society of Cardiology guidelines for the diagnosis of AHF (15): plasma natriuretic peptide level (BNP ≥ 100 pg/ml) (Class I, Level A), a 12-lead electrocardiogram (Class I, Level C), laboratory measurements [troponins, blood urea nitrogen (BUN), creatinine, sodium, potassium, glucose, liver function, and complete blood counts] (Class I, Level C), and echocardiography (Class I, Level C). In addition, patients who presented with new or exacerbated symptoms and signs of HF, and met the Framingham Criteria (16), were also eligible. In brief, the Framingham criteria require two major criteria, or one major and two minor criteria: the major criteria include orthopnoea or paroxysmal nocturnal dyspnoea, neck vein distention, rales, cardiomegaly, acute pulmonary edema, S3 gallop, increased venous pressure, prolongation of circulation time, and hepatojugular reflux; the minor criteria include ankle edema, night cough, dyspnoea on exertion, hepatomegaly, tachycardia, and weight loss. An experienced physician assessed all patients at the emergency department within 30 min of admission.

Exclusion criteria were: (I) pulmonary embolism; (II) death during the hospital stay; (III) acute coronary syndrome; (IV) bradycardia requiring pacemaker implantation; (V) estimated glomerular filtration rate (eGFR) < 15 ml/(min·1.73 m^2^); (VI) planned dialysis or has entered maintenance dialysis; (VII) had a history of large organ transplantation; (VIII) participated in a drug treatment study within the past 30 days or before; (IX) pregnant and lactating women.

This work has been carried out in accordance with the Declaration of Helsinki (2000) of the World Medical Association. This study protocol was approved by the Ethics Review Committee of Shanghai General Hospital (2022KY031). The Institutional Review Board waived the requirement for written informed consent because this study was a retrospective observational study.

### Definition of WRF degree and BNP grouping

WRF was divided into two grades according to the different degrees of Scr changes during hospitalization: non-severe WRF (nsWRF) and severe WRF (sWRF). Based on the commonly used definition of WRF (Scr increase ≥0.3 mg/dl from admission) (6), nsWRF was defined as the increase of Scr during hospitalization by ≥0.3 mg/dl and <0.5 mg/dl compared with that on admission; sWRF was defined as the Scr continued to increase by ≥0.5 mg/dl compared with admission. Non-WRF (nWRF) was defined as an increase in Scr of <0.3 mg/dl during hospitalization compared with admission. We divided patients into high and low BNP groups based on the median BNP at discharge to evaluate the effect of different degrees of WRF on 1-year death in AHF under different BNPs at discharge. The low BNP group were those with BNP levels lower than the median at discharge, while the high BNP group included those with BNP levels higher than the median BNP at discharge.

### Statistical analysis

For numerical variables with normal distribution, the mean ± standard deviation (SD) was used to describe between-group differences; these data were assessed by t-test or one-way ANOVA. When the data of numerical variables were skewed, the median was used, and interquartile range [M(QR)] descriptions and comparisons between groups were performed using the Mann-Whitney U rank-sum test or the Kruskal-Wallis rank-sum test. Categorical variable data were described by the number of cases and frequency [n (%)], and comparisons between groups were performed using the Chi-square test or the exact probability method. Kaplan-Meier survival analysis was used to analyze the effects of discharge plasma BNP level and different degrees of WRF on the 1-year survival rate, and the log-rank test was performed. The same method was used to analyze the effect of different degrees of WRF on 1-year mortality under different BNP levels at discharge. Univariate and multivariate COX proportional hazards regression models were used to analyze whether low BNP value at discharge or different degrees of WRF were associated with mortality, as well as testing for an interaction between the two. In multivariate analysis, differences in age, sex, pulmonary crackles, peripheral edema, systolic blood pressure and hemoglobin on admission, serum sodium, BUN, and Scr at discharge, and history of admission for ischemic heart disease, atrial fibrillation, hypertension, diabetes, chronic kidney disease were adjusted based on prior studies (17–19). Two-sided *P* < 0.05 was considered statistically significant. Data were analyzed by IBM SPSS Statistics 19.

## Results

Table 1 shows the basic characteristics of patients at admission and discharge. Among 445 patients who initially met the inclusion requirements, 5 patients who could not be reached by telephone were excluded. Ultimately, we analyzed the survival status of 440 patients within 1 year after discharge. The median age was 74 years; there were 263 males (59.8%). In addition, 252 patients had a history of ischemic heart disease (57.3%), 161 had a history of atrial fibrillation (36.6%), 184 patients a history of diabetes (41.8%), 356 patients a history of hypertension (80.9%), and 106 patients had a history of chronic kidney disease (23.9%).

**Table 1:** Baseline characteristics among different groups.

Variable	Totle (*n* = 440)	nWRF (*n* = 281)	nsWRF (*n* = 75)	sWRF (*n* = 84)	*P* value	Low BNP (*n* = 220)	High BNP (*n* = 220)	*P* value
Age (year)	74 (19)	74 (19)	77 (19)	75 (22)	0.597	77 (17)	72 (19)	0.013
Male, *n* (%)	263 (59.8)	162 (57.7)	53 (70.7)	48 (57.1)	0.107	131 (59.5)	132 (60.0)	0.923
Hospital time (day)	11 (9)	10 (6)	14 (14)	14 (16)	<0.001	11 (9)	11 (10)	0.894
Systolic BP (mmHg)	136 ± 25	135 ± 23	134 ± 28	140 ± 30	0.236	139 ± 24	133 ± 26	<0.001
LVEF	43.9 ± 12.5	44.3 ± 12.7	43.5 ± 13.9	42.8 ± 10.8	0.613	44.6 ± 12.5	43.1 ± 12.6	0.188
Pulmonary crackles, *n* (%)	201 (46.7)	115 (40.9)	35 (46.7)	51 (60.7)	0.006	104 (47.3)	97 (44.1)	0.503
Peripheral edema, *n* (%)	126 (28.6)	79 (28.1)	21 (28.0)	26 (31.0)	0.872	53 (24.1)	73 (33.2)	0.035
History of IHD, *n* (%)	252 (57.3)	156 (55.5)	51 (68.0)	45 (53.6)	0.114	124 (56.4)	128 (58.2)	0.7
History of atrial fibrillation, *n* (%)	161 (36.6)	107 (38.1)	27 (36.0)	27 (32.1)	0.608	82 (37.3)	79 (35.9)	0.767
History of hypertension, *n* (%)	356 (80.9)	217 (77.2)	65 (86.7)	74 (88.1)	0.032	177 (80.5)	179 (81.4)	0.808
History of diabetes mellitus, *n* (%)	184 (41.8)	108 (38.4)	39 (52.0)	37 (44.0)	0.096	87 (39.5)	97 (44.1)	0.334
History of CKD, *n* (%)	106 (23.9)	27 (9.6)	26 (34.7)	52 (61.9)	<0.001	42 (19.1)	63 (28.6)	0.019
**In hospital**								
Hemoglobin (g/L)	119 ± 25	124 ± 22	115 ± 26	105 ± 27	<0.001	120 ± 25	118 ± 25	0.385
Sodium (mmol/L)	140.0 ± 4.5	140.1 ± 4.0	140.0 ± 4.6	140.0 ± 5.9	0.986	140.2 ± 4.8	139.9 ± 4.2	0.474
Potassium (mmol/L)	4.0 ± 0.6	3.9 ± 0.5	4.0 ± 0.6	4.3 ± 0.9	<0.001	3.9 ± 0.6	4.0 ± 0.7	0.106
BUN(mmol/L)	8.50 (5.6)	7.50 (3.64)	10.09 (5.60)	15.98 (10.57)	<0.001	7.80 (4.40)	9.30 (6.15)	<0.001
Scr (μmol/L)	95.0 (61.4)	84.6 (30.2)	125.3 (51.1)	208.9 (177.6)	<0.001	92.5 (47.3)	100.9 (76.1)	0.04
BNP (pg/ml)	943 (912)	818 (791)	1,044 (972)	1,152 (839)	0.002	586 (627)	1,193 (970)	<0.001
**In-hospital treatment**								
Inotropes, *n* (%)	58 (13.2)	32 (11.4)	16 (21.3)	10 (11.9)	0.072	28 (12.7)	30 (13.6)	0.778
Intravenous loop diuretics, *n* (%)	397 (90.2)	248 (88.3)	70 (93.3)	79 (94.0)	0.178	200 (90.9)	197 (89.5)	0.630
**At discharge**								
Sodium (mmol/L)	139.4 ± 4.9	139.2 ± 4.1	139.3 ± 4.6	140.2 ± 7.1	0.254	139.6 ± 4.6	139.2 ± 5.1	0.417
BUN (mmol/L)	8.00 (5.55)	7.05 (3.47)	9.86 (6.61)	13.04 (14.41)	<0.001	7.50 (4.98)	9.00 (6.15)	<0.001
Scr (μmol/L)	91.8 (51.3)	80.0 (31.8)	114.0 (52)	175.9 (169.4)	<0.001	89.0 (41.8)	96.0 (68.0)	0.017
BNP (pg/ml)	464 (626)	437 (546)	533 (698)	700 (778)	0.010	228 (168)	850 (588)	<0.001
**Medication at dischrage**								
ACEI/ARBs, *n* (%)	236 (53.6)	152 (54.1)	40 (53.3)	44 (52.4)	0.961	117 (53.2)	119 (54.1)	0.848
Beta-blockers, *n* (%)	300 (68.2)	186 (66.2)	53 (70.7)	61 (72.6)	0.475	158 (71.8)	142 (64.5)	0.101
Loop diuretics, *n* (%)	308 (70.0)	199 (70.8)	55 (73.3)	54 (64.3)	0.408	147 (66.8)	161 (73.2)	0.145
Aidosterone antagonists, *n* (%)	203 (46.1)	134 (47.7)	37 (49.3)	32 (38.1)	0.251	99 (45.0)	104 (47.3)	0.633

BP, blood pressure; LVEF, left ventricular ejection fraction; IHD, ischemic heart disease; CKD, chronic kidney disease; BUN, blood urea nitrogen; Scr, serum creatinine; BNP,B-type natriuretic peptide.

The median BNP at discharge was 464 pg/ml. WRF occurred in 159 patients (36.1%), including 75 cases of nsWRF and 84 cases of sWRF. Within 1 year after discharge, 89 patients died, resulting in a mortality rate of 20.2%. Patients in the sWRF group spent more time (days) in the hospital and had higher rates of crackles, a history of CKD, hypertension, and higher potassium at admission (*P* < 0.05). Intravenous loop diuretics were more frequently used in the sWRF group (*P* = 0.178). Patients in the nWRF group had higher hemoglobin on admission but lower BUN, Scr, and BNP at admission and discharge (*P* < 0.05). Patients with low BNP levels were older with higher SBP (*P* < 0.05). The incidence of CKD and edema was higher in patients with high BNP levels (*P* < 0.05). They had higher BUN, Scr, and BNP at admission and discharge (*P* < 0.05).

### Effect of BNP level on 1-year death at discharge

**Figure 1:**
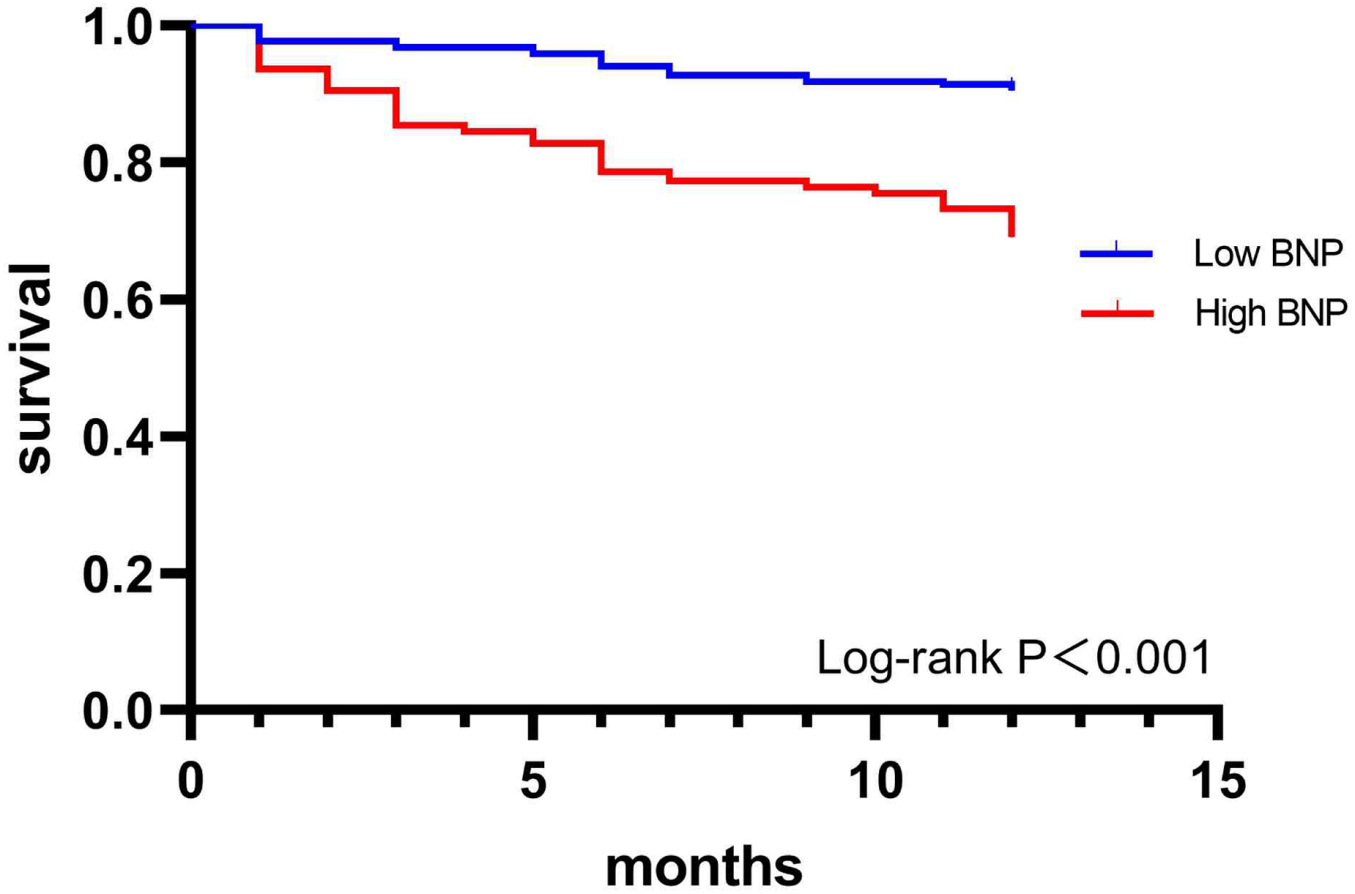
1-year survival curve of AHF patients in the high BNP group and low BNP group.

The one-year mortality (30.9%) of the high BNP group was higher than that of the low BNP group (9.5%) (*P* < 0.001). The Kaplan-Meier survival curves of the two groups are shown in Figure 1, and the low BNP group was associated with better outcomes (*P* < 0.001).

### Effect of 1-year mortality in WRF

**Figure 2:**
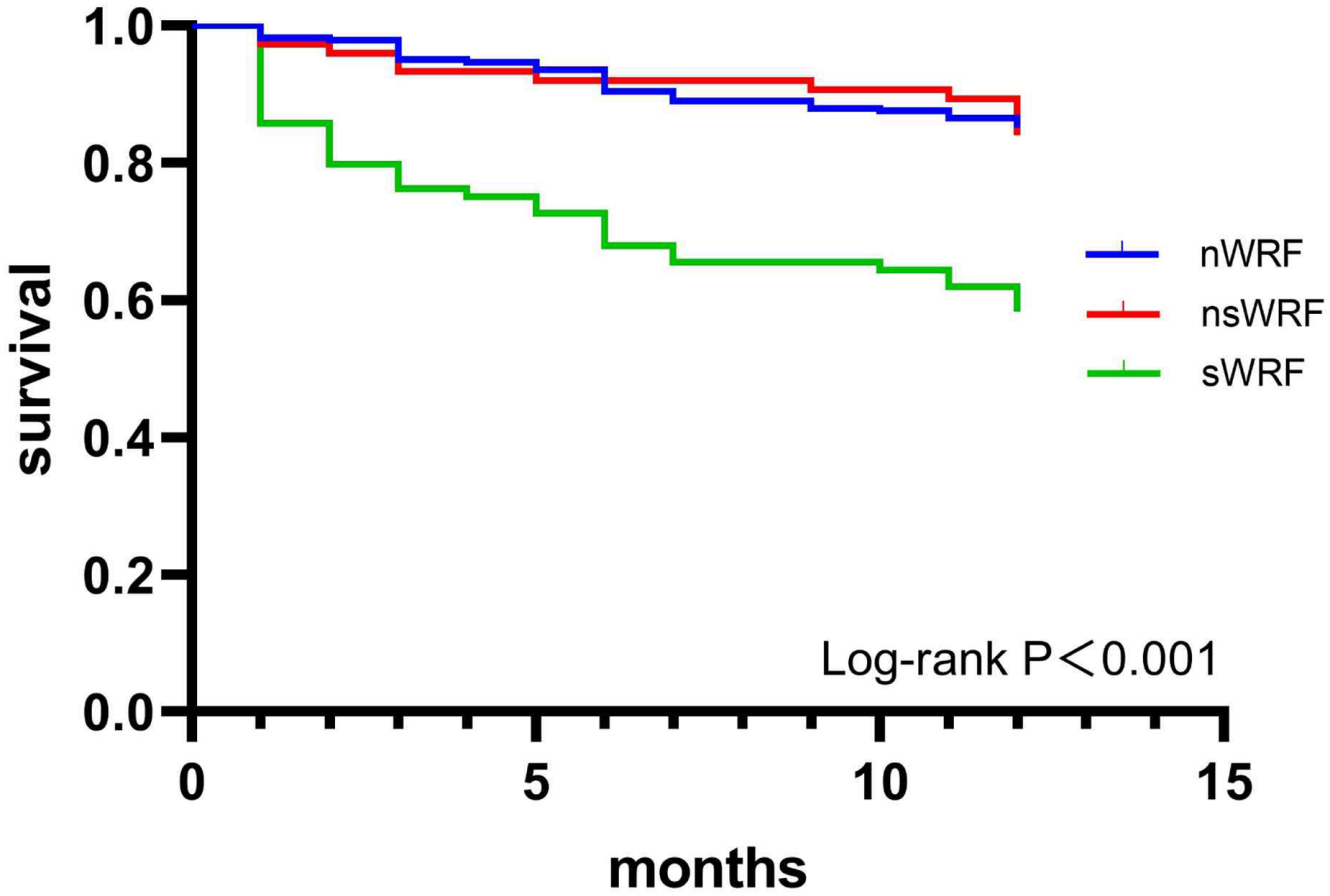
1-year survival curve of patients in nWRF, nsWRF, and sWRF groups.

The mortality of the sWRF group (41.7%) was higher than that of the nWRF (14.9%) group and the nsWRF group (16.0%) (*P* < 0.001), while there was no difference in mortality between the nWRF group and nsWRF group (*P* = 0.821) (Table 2). The Kaplan-Meier survival curves of the three groups are shown in Figure 2, and sWRF was associated with worse outcomes (*P* < 0.001).

**Table 2:** Comparison of 1-year mortality among different groups.

Group	Total	Number of death	mortality (%)	* *
**BNP group**				
Low BNP	220	21	9.5	*χ*^2 ^= 31.11
High BNP	220	68	30.9	*P *< 0.001
WRF phenotype				
nWRF	281	42	14.9	*χ*^2 ^= 29.62
nsWRF	75	12	16	*P *< 0.001
sWRF	84	35	41.7	

The comparison of nWRF group and nsWRF group, *χ*^2 ^= 0.05, *P* = 0.821; The comparison of nsWRF group and sWRF group, *χ*^2 ^= 11.53, *P* < 0.001; The comparison of nWRF group and sWRF, *χ*^2 ^= 27.74, *P* < 0.001.

**Figure 3:**
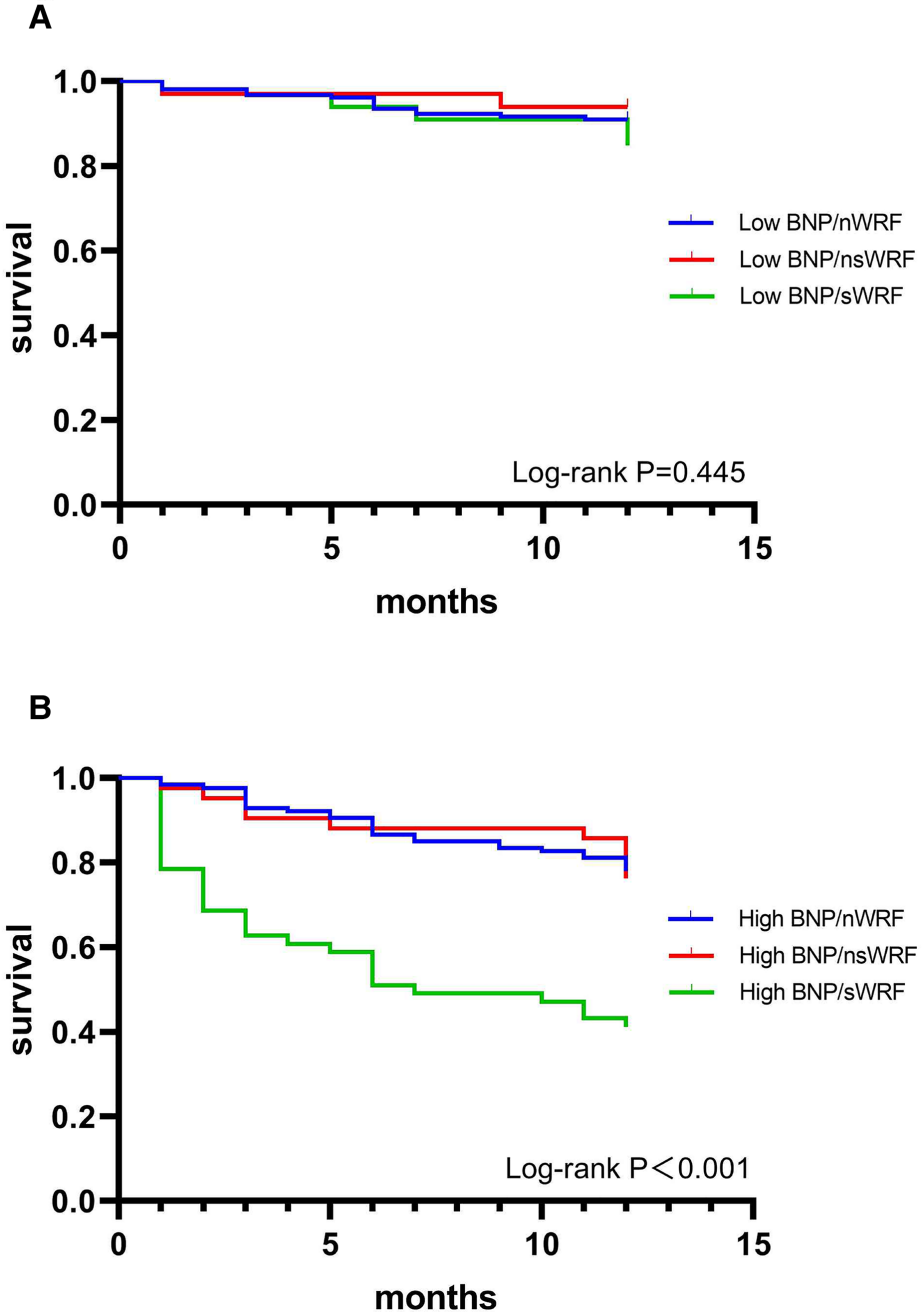
1-year survival curves of nWRF, nsWRF, and sWRF in patients with low BNP (**A**) and high BNP (**B**).

In the low BNP group, the 1-year morality of different degrees of WRF is shown in Table 3. There was no significant difference among the nWRF, nsWRF, and sWRF (*P* = 0.489). The Kaplan-Meier survival curves of the three groups are shown in Figure 3A, and the 1-year survival showed no significant difference between the three groups (*P* = 0.446). In the high BNP group, the differences among the three groups in morality were statistically significant (*P* < 0.001). The mortality of sWRF was significantly different from the other two groups (*P* < 0.01), but the mortality of nsWRF was not significantly different from that of nWRF (*P* = 0.813) in the high BNP group, as shown in Table 3. The Kaplan-Meier survival curves of different degrees of WRF are shown in Figure 3B, and sWRF in the high BNP group had the worst clinical outcomes.

**Table 3:** Effect of discharged BNP and worsening renal function on 1-year mortality.

	Low BNP			High BNP			* *
	Total	Number of death	Mortality (%)	Total	Number of death	Mortality (%)	
nWRF	154	14	9.1	127	28	22	*χ^2 ^*= 9.19, *P *< 0.001
nsWRF	33	2	6.1	42	10	23.8	*χ^2 ^*= 4.33, *P *< 0.03
sWRF	33	5	15.2	51	30	58.8	*χ^2 ^*= 15.72, *P *< 0.001
	Fisher's exact test *P *= 0.489			*χ*^2 ^= 24.27, *P *< 0.001			——

In the high BNP group,the comparison of nWRF group and nsWRF group, *χ*^2 ^= 0.06, *P *= 0.813; The comparison of nsWRF group and sWRF group, *χ*^2^ = 11.52, *P* = 0.001; The comparison of nWRF group and sWRF, *χ*^2^ = 22.40, *P* < 0.001.

**Figure 4:**
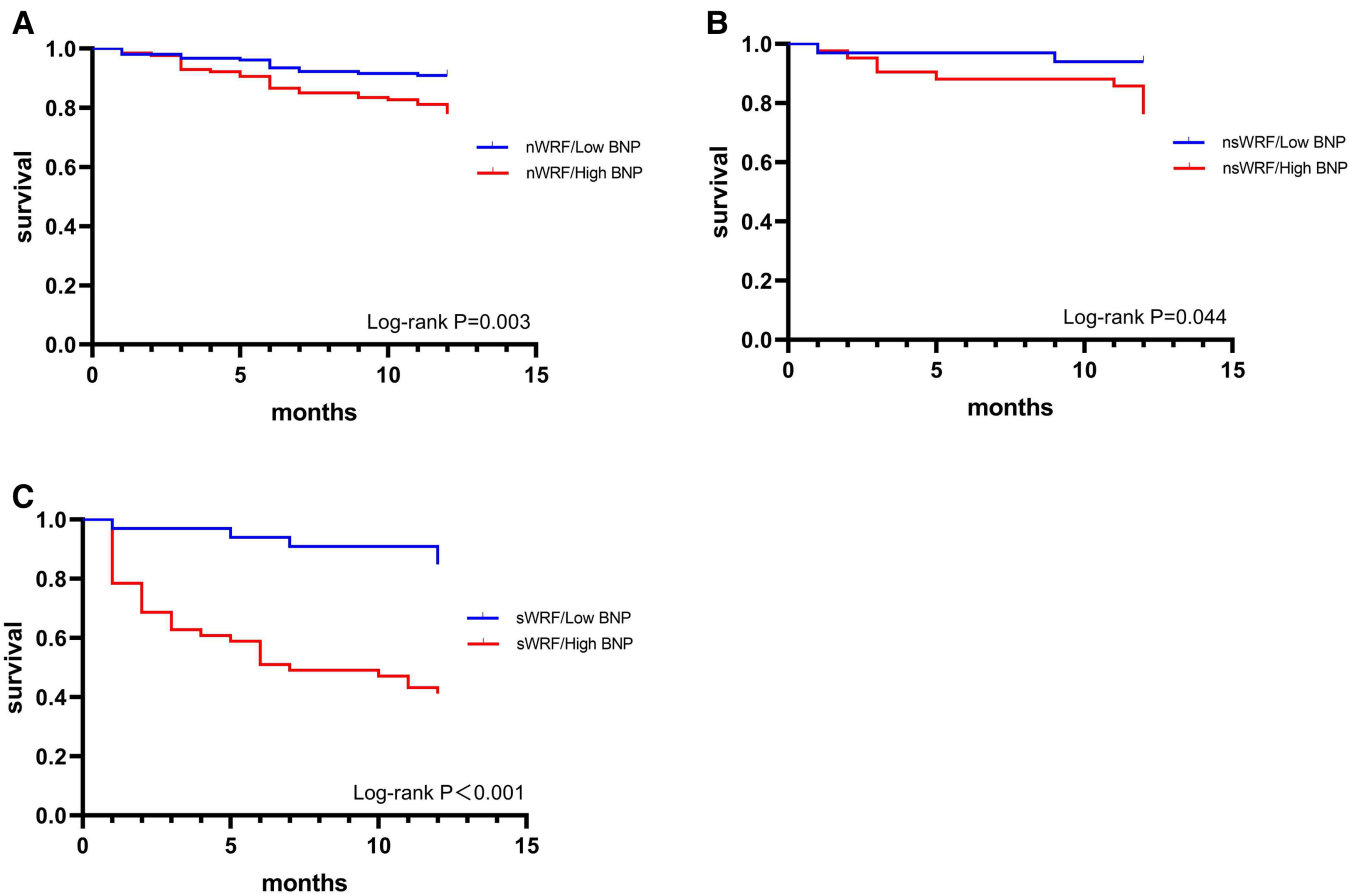
1-year survival curve of low BNP and high BNP group in nWRF (**A**), nsWRF (**B**), and sWRF (**C**).

According to nWRF, nsWRF, and sWRF, three groups were set to compare the 1-year morality of high BNP and low BNP groups. In each WRF group, mortality was higher in the high BNP group than in the low BNP group (Table 3). The Kaplan-Meier survival curves in Figures 4A–C showed that in each WRF group, the high BNP group always had the worse outcome in contrast to the low BNP group.

Results from univariate and multivariate Cox proportional hazards models for 1-year morality are shown in Table 4. In a multivariate COX proportional hazards model, the low BNP group was associated with decreased mortality [hazard ratio(HR), 0.265; 95% confidence interval (CI), 0.162%0.434; *P* < 0.001]. sWRF resulted as an independent predictor of mortality (HR, 2.838; 95% CI, 1.756–4.589; *P* < 0.001). Also, there was no increased 1-year risk of death in nsWRF compared to nWRF(HR, 0.886; 95% CI, 0.461%1.703; *P* = 0.717). There was a significant interaction between the low BNP group and sWRF (HR, 0.225; 95%CI, 0.055%0.918; *P* = 0.038).

**Table 4:** Univariate and multivariate Cox proportional hazards regression analysis of 1-year mortality in patients with AHF.

	Univariate analysis		Multivariate analysis	
	HR (95%CI)	*P* value	Adjusted HR (95%CI)	*P* value
low BNP group	0.275 (0.169,0,449)	<0.001	0.265 (0.162,0.434)	<0.001
nsWRF	1.067 (0.562,2.027)	0.842	0.886 (0.461,1.703)	0.717
sWRF	3.400 (2.170,5.329)	<0.001	2.838 (1.756,4.589)	<0.001
Interaction between low BNP group and different degrees of WRF	_	_	_	0.041
Interaction between low BNP group and nsWRF	_	_	0.488 (0.195,1.224)	0.126
Interaction between low BNP group and sWRF	_	_	0.225 (0.055,0.918)	0.038

HR, hazard ratio; CI, confidence interval; Multivariate analysis adjusted the factors that may affect 1-year mortality.

## Discussion

The present study analyzed the effect of the degree of WRF on the long-term prognosis of AHF patients under different BNP levels at discharge. The key findings of this study are: (I) in AHF patients, nsWRF was not associated with 1-year all-cause mortality, while sWRF was associated with mortality and resulted as an independent predictor of AHF prognosis; (II) low-level BNP at discharge, regardless of the occurrence of WRF, was associated with lower mortality and resulted as an independent predictor of long-term prognosis in AHF; (III) low-level BNP at discharge could reduce the adverse effects of sWRF on prognosis.

In the present study, we divided the severity of WRF to assess whether the degrees of WRF have different effects on AHF's prognosis. Since the definition of WRF has not yet been unified, the increase of Scr concentration was utilized as the basis for grading the severity of WRF. COX multivariate regression analysis showed that compared to the nWRF, sWRF could increase the mortality risk of AHF patients. It also resulted as an independent predictor for mortality, which is consistent with previous studies (17, 20). In addition, nsWRF did not increase the risk of mortality in AHF compared to the nWRF. Previous studies have used Scr increase ≥0.3 mg/dl as the definition of WRF to study prognosis, but the results for the long-term prognosis of AHF were conflicting (17, 20, 21). The contradiction may be due to the different degrees of increased Scr in these studies. The proportion of patients with Scr may increase ≥0.3 mg/dl and <0.5 mg/dl was different in each study. This group is the nsWRF group that we studied. We speculate that this could be caused by neurohormonal or hemodynamic abnormalities, which lead to reversible renal dysfunction and does not cause real kidney injury (22, 23). Considering the prognosis of renal function in patients with nsWRF and sWRF after discharge remains unclear, as well as the eventual existence of differences in the long-term effects on kidney function, future studies should determine the long-term prognosis of nsWRF and sWRF based on the changes in sensitive renal function markers such as Scr, cystatin C, and neutrophil gelatinase-associated lipocalin (NGAL) after discharge.

Although sWRF resulted as an independent predictor of mortality in AHF, sWRF was only associated with mortality in the group with high BNP levels and interacted with a low BNP level, showing that BNP value at discharge assessed with creatinine can differentiate WRF with relevant prognosis from non-relevant WRF in AHF. Thus, the study results imply that sWRF occurring in hospitals should be interpreted in conjunction with BNP at discharge in patients with AHF. Metra et al. (13) used data from the PROTECT study (Placebo-Controlled Randomized Study of the Selective A1 Adenosine Receptor Antagonist Rolofylline for Patients Hospitalized With Acute Decompensated Heart Failure and Volume Overload to Assess Treatment Effect on Congestion and Renal Function) to investigate whether the degree of congestion affects the prognostic significance of WRF in AHF and found that among patients with an increase in Scr of 26.5 μmol/L during their hospital admission, only those with congestion had a higher risk of death. Similarly, McCallum et al. (24) demonstrated that when kidney function declines in a hospital, acute decompensated heart failure appears benign as long as decongestion is achieved. Our study reinforces the importance of congestion in terms of the prognostic relevance of sWRF.

Correct assessment of the extent of decongestion is critical in recognizing the prognostic meaning of sWRF, but it is also challenging. BNP has been used to assess patients' volume status because of its objective and convenient measurement and has been associated with prognosis in HF patients (25, 26). A previous study suggested that a percent BNP reduction from admission to discharge independently predicts worse outcomes in AHF (27). Nevertheless, Hamatani et al. (28) demonstrated that the change in the ratio of BNP at discharge to admission is often used to predict mortality in AHF but is not as accurate as the value of BNP at discharge. However, the exact effect of BNP reduction at discharge on the risk of death is clinically unclear for AHF patients. Studies have shown that the threshold of BNP at discharge ≤ 250 pg/ml can significantly reduce AHF patients' mortality and readmission rate (25). However, achieving this threshold for patients with high BNP values at admission with decongestion therapy such as diuretics is also difficult. Intermediate threshold BNP may be more clinically useful (29). Also, there is no clear intermediate threshold BNP to judge AHF patient prognosis. This study used the median BNP value at discharge (464 pg/ml) to group patients and confirmed results of previous studies; regardless of whether WRF occurred, low BNP value(<464 pg/ml) at discharge was associated with a lower 1-year risk of death.

The reason why sWRF has a different effect on prognosis under different fluid volume loads may be because pathogeneses leading to sWRF are different under different fluid volume loads. Renal vascular congestion may lead to sWRF due to increased renal interstitial pressure, renal tubule damage, and hypoxic damage to the renal cortex, affecting prognosis (30). Although aggressive decongestion therapy increases the incidence of sWRF, it is mainly transient kidney damage, which has no adverse effect on prognosis. A study using NGAL to assess tubular damage showed that WRF under decongestion appears to be associated with hemodynamic changes independent of tubular damage (31). Clinically, when renal function worsens in AHF patients, cardiologists and nephrologists often have different opinions on the next decongestion therapy plan. Cardiologists worry that insufficient decongestion increases the risk of death, while nephrologists are concerned that excessive decongestion might increase the risk of death by exacerbating kidney damage. Our study showed that in the absence of WRF, it is better to reduce the body volume load of patients at discharge as much as possible for long-term prognosis, and the occurrence of nsWRF should not be particularly emphasized to ensure renal perfusion and reduce decongestion therapy. However, only when sWRF occurs, it is necessary to pay attention not to excessively reduce body volume whilst focusing on renal function in order to timely detect and treat kidney damage. Reducing body volume load should not be regarded as the only goal of improving the prognosis.

Previous studies found that WRF with higher BNP levels at discharge had a worse prognosis (32, 33). This study further found that regardless of the degree of WRF, 1-year mortality was higher in patients with higher BNP levels at discharge. BNP had a better prognostic value than WRF. Therefore, WRF may be a prognostic parameter in certain subgroups of patients (e.g., with high BNP). When WRF occurs, the determination of BNP helps to assess the congestion status and prognosis of patients at this time and can further stratify such risk groups, which is helpful for clinicians to perform different treatment measures promptly. Our study showed that when BNP is controlled below 464 pg/ml at discharge while allowing an increase in Scr within 0.5 mg/dl during hospitalization, the prognosis of AHF patients may improve.

## Study limitations

First, this is a single-center retrospective study. Unmeasured variables and missing data may affect the results. Second, this study did not explore other variables influencing BNP reduction and the relationship between WRF. Third, our study only analyzed changes in renal function during hospitalization, and data on subsequent changes in renal function after discharge are lacking. As these data may be more helpful in identifying whether there is actual renal impairment, they should also be included in further analyses. Fourth, we classified patients using the median BNP at discharge as a cutoff value. Although plasma BNP is a reliable marker for measuring congestion in HF patients, our classification may not fully identify congestion patients.

## Conclusions

nsWRF does not increase the 1-year risk of all-cause mortality in AHF patients, whereas sWRF does. In addition, a low BNP value at discharge is associated with a lower 1-year risk of all-cause mortality in AHF patients, while a low BNP value at discharge could mitigate the adverse effects of sWRF on the prognosis.

## Data Availability

The original contributions presented in the study are included in the article/Supplementary Material, further inquiries can be directed to the corresponding author/s.
